# Diagnostic and prognostic value of myocardial work indices in evaluating cardiac function damage in active acromegaly patients

**DOI:** 10.1530/EC-25-0478

**Published:** 2026-01-12

**Authors:** Rong Huang, Pengyuan Zhang, Wei He, Jiewen Jin, Yang Peng, Yanbing Li, Haipeng Xiao, Fengjuan Yao, Hai Li

**Affiliations:** ^1^ Department of Endocrinology, The First Affiliated Hospital, Sun Yat-Sen University, Guangzhou, China; ^2^ Department of Medical Ultrasonics, The First Affiliated Hospital, Sun Yat-Sen University, Guangzhou, China; ^3^ Department of Radiology, The First Affiliated Hospital, Sun Yat-Sen University, Guangzhou, China; ^4^ Department of Endocrinology, Guizhou Hospital of The First Affiliated Hospital of Sun Yat-sen University, Guizhou, China

**Keywords:** acromegaly, speckle tracking echocardiography, myocardial work, left ventricular pressure-strain loop

## Abstract

**Objective:**

Strain imaging serves as a sensitive marker for detecting early subclinical myocardial systolic dysfunction. The purpose of this study was to evaluate the diagnostic and prognostic value of myocardial work indices in assessing subclinical myocardial systolic dysfunction in active acromegaly patients.

**Methods:**

27 active acromegaly patients and 27 healthy controls matched for age, sex, height, weight, body mass index and body surface area were included in the study. Active acromegaly was diagnosed based on elevated serum insulin-like growth factor 1 (IGF-1) (>1× upper limit of normal) or insufficient GH suppression (nadir ≥0.4 ng/mL) during an OGTT. All participants underwent two-dimensional speckle-tracking echocardiography (2D-STE) for the assessment of cardiac function. STE extracted the corresponding strain parameters (such as global longitudinal strain (GLS), global circumferential strain and global radial strain) and work parameters (such as global work index, global constructive work, global wasted work (GWW) and global work efficiency (GWE)) by analyzing the motion (strain)–velocity (strain rate) of two or more local myocardial segments, combined with the left ventricular non-invasive pressure estimation technique. At the same time, correlation analysis was used to explore the factors affecting GWW and GWE in the acromegaly group.

**Results:**

In comparison with the control group, conventional echocardiography revealed that acromegaly patients did not exhibit a significant difference in left ventricular ejection fraction (60.3 ± 3.7 vs 59.1 ± 4.8, *P* = 0.312), a commonly used index to evaluate ventricular systolic function, STE showed that there was no significant difference in GLS (−18.3 ± 2.4 vs −17.4 ± 2.9, *P* = 0.514) between the control group and the acromegaly group. However, significant differences can be found in GWW (44.8 ± 31.1 vs 80.6 ± 75.6, *P* = 0.027) and GWE (97.0 ± 1.8 vs 95.0 ± 3.8, *P* = 0.020), and a significant correlation was observed between myocardial work parameters and 1.5 × ULN IGF-1.

**Conclusion:**

GWW and GWE are sensitive markers for identifying subclinical myocardial systolic dysfunction, suggesting their potential as early markers for detecting subclinical myocardial systolic dysfunction in active acromegaly patients.

## Introduction

Acromegaly is a rare disease characterized by excessive secretion of growth hormone (GH) and the peripheral target hormone IGF-1 (1, 2). Data show that although the mortality rate of cardiovascular disease in acromegaly patients has decreased from 44 to 23%, it is still one of the leading causes of death in this population, second only to malignant tumors (3). Cardiovascular complications in acromegaly patients include cardiomyopathy (59%), heart failure (11%) and coronary heart disease (6%) (4). Cardiovascular damage in acromegaly starts with structural changes (such as left ventricular hypertrophy (LVH)) and then develops diastolic dysfunction, mainly manifested by increased E/E′ ratio and prolonged isovolumetric relaxation time. Long-term uncontrolled diastolic dysfunction ultimately leads to myocardial remodeling (such as ventricular dilation and fibrosis), which in turn leads to a decline in systolic function, with GH/IGF-1 levels being the main driving factor (5, 6). In active acromegaly patients, cardiovascular damage is more pronounced, with LVH and diastolic dysfunction being the most common myocardial changes detected by echocardiography (7). This may progress to systolic dysfunction and myocardial ischemia, contributing to adverse outcomes such as coronary heart disease and heart failure (8, 9). Early subclinical changes in myocardial function in acromegaly patients are potentially reversible with treatment, emphasizing the importance of early diagnosis and timely intervention to prevent irreversible damage (10, 11). However, traditional Doppler echocardiography has limitations in accurately identifying early subclinical myocardial structural and functional changes in patients with acromegaly. The ‘gold standard’ for detecting myocardial contractile dysfunction, left ventricular ejection fraction (LVEF), is often within the normal range, which may delay treatment and lead to progressive deterioration of myocardial function. Therefore, there is an urgent need for a more sensitive detection technique (12).

LVEF is the main index to evaluate ventricular systolic function by Doppler echocardiography. However, LVEF cannot promptly identify early subclinical myocardial damage (13). Speckle tracking echocardiography (STE) has emerged as a technique for evaluating the segmental and global systolic function of the ventricular wall by tracking the speckle signals generated by ultrasonic waves with wavelengths less than or equal to the size of myocardial tissue (14). Compared with the conventional echocardiography, STE reduces the inter-observer and intra-observer variability, overcomes defects such as angle dependence and noise interference and makes non-invasive quantitative analysis of myocardial segments and overall function (14, 15). However, STE is load dependent, and the value of myocardial strain decreases with the increase in afterload, which leads to misjudgment of myocardial contraction (16). Myocardial work (MW), combined with speckle tracking ultrasound and afterload measurement, can comprehensively and objectively evaluate the changes of MW and myocardial oxygen consumption, which may have great potential in clinical applications (17).

Global longitudinal strain (GLS) detected by STE has been shown to represent a sensitive parameter for subclinical myocardial systolic dysfunction in patients with Cushing’s syndrome and can be used for an early diagnosis and evaluation of myocardial injury in patients with normal LVEF (18). Therefore, we speculated whether acromegaly, which also damages myocardium, might exhibit similar changes. At present, early markers of subclinical myocardial systolic dysfunction in active acromegaly patients are unclear. The purpose of this study is to explore the changes of myocardial structure and function in active acromegaly patients, which could serve as potential markers of subclinical myocardial systolic dysfunction.

## Materials and methods

### Study population

We evaluated 34 active acromegaly patients who were diagnosed and treated in the Department of Endocrinology, a university from August 2021 to March 2022, of which 7 patients were excluded due to poor echocardiographic image quality, which might have compromised the accuracy of MW parameter assessment. This study was designed as a single-center, retrospective analysis.

The diagnostic criteria of acromegaly were typical clinical manifestations, increased serum IGF-1 levels matched for age and sex, the failure of GH to be suppressed to below 1 ng/mL during the 75 g oral glucose tolerance test (OGTT) and positive pituitary MRI results (19). The diagnostic criteria of active acromegaly were as follows: i) random GH ≥ 1 μg/L or lowest GH ≥ 0.4 μg/L after OGTT, ii) elevated IGF-1 level (>100% upper limit of normal) and iii) being clinically active as assessed by researchers (20, 21). All patients are in the active phase of the disease. Among the 27 patients, 13 (48.1%) were treatment-naive, 8 (29.6%) experienced recurrence after remission, and 6 (22.3%) failed to achieve biochemical control after treatment.

The control group consisted of 27 healthy individuals matched for age, sex, height, weight, body mass index (BMI) and body surface area (BSA). The exclusion criteria for both groups are as follows: i) coronary artery disease, heart failure, arrhythmia, stroke/transient cerebral ischemia, peripheral artery disease, chronic renal failure, respiratory failure and pregnancy; ii) age <18 years old.

Our research was approved by the Human Ethics Committee of a university and was in line with the Declaration of Helsinki. All subjects signed the informed consent form.

### Echocardiography

Standard two-dimensional transthoracic echocardiographic images were recorded using a Vivid E9 echocardiography system (General Electric Vingmed Ultrasound, USA). Multiple continuous cardiac cycles of standard echocardiographic views were collected, and the images were digitally stored and analyzed offline using proprietary software (EchoPAC 202; GE Vingmed Ultrasound). The cardiac diameter and ventricular wall thickness were measured from the parasternal long axis section. Left atrial and ventricular volumes were evaluated by biplane Simpson and area-length methods. The peak early-diastolic mitral valve flow velocity (E) and end-diastolic mitral valve flow velocity (A) were measured from the apical four-chamber view, and the E/A ratio was used to represent left ventricular diastolic function. The peak velocity (e′) of the mitral annulus in early diastole is measured using pulsed wave tissue Doppler technology, and the calculated E/e′ can be used to indicate left ventricular filling pressure. Meanwhile, LVEF calculated using the biplane Simpson method is a commonly used parameter to judge left ventricular systolic function.

### Myocardial work evaluation

While GLS obtained by speckle-tracking echocardiography (STE) offers high sensitivity and good reproducibility, its clinical utility is constrained by an inherent limitation of load dependency. MW parameters, in contrast, integrate left ventricular pressure–strain relationships, thereby compensating for the afterload dependency inherent in GLS. By integrating STE with the estimated left ventricular pressure curve, a non-invasive pressure–strain loop (PSL) of the left ventricle was constructed to represent MW. The estimated left ventricular pressure curve of this non-invasive measurement introduced by Russell *et al.* was produced by combining peripheral systolic blood pressure with the time of cardiac events (including isovolumetric systole, ejection phase and isovolumetric relaxation), which could be obtained by measuring the opening and closing times of the heart valve. The systolic blood pressure (SBP) measured by the cuff arm was used to represent the left ventricular absolute systolic blood pressure (LVSP) (17). Through STE, trace the left ventricular endocardial border delineated by small and stable myocardial footprints or spots within a predefined region of interest in apical standard two-chamber, three-chamber and four-chamber views, and measure ventricular strain parameters in different views. The strain data and the blood pressure data measured by the cuff arm blood pressure were then used to construct a left ventricular PSL curve, which was standardized and adjusted according to the duration of the different stages of the cardiac event (isovolumetric systole, ejection phase and isovolumetric relaxation).

MW was measured during left ventricular systolic ejection, starting when the mitral valve was closed and ending when the mitral valve was open. Global work index (GWI) is expressed as the overall work in the left ventricular PSL region, that is, the sum of MW during mechanical systole (from mitral valve closure to mitral valve opening) plus isovolumetric systole and isovolumetric relaxation. Global constructive work (GCW) represents the work performed by myocardial segments during the shortening of systole and the lengthening of isovolumetric diastole, that is, the work done to effectively promote left ventricular ejection and relaxation. Global wasted work (GWW) is defined as the work performed during systole prolongation or isovolumetric diastole shortening, that is, an ineffective contribution to left ventricular ejection or relaxation. Global work efficiency (GWE) represents the percentage of systolic and diastolic effective work done in the total left ventricular work.

### Statistical analysis

The demographic characteristics and clinical data of the subjects were statistically analyzed using SPSS 25.0 software (IBM, USA). The data were tested for normality based on histograms and scatter plots combined with the Shapiro–Wilk test. For normally distributed continuous numerical variables, they were expressed as mean ± SD, and the Student-*t* test was used to analyze the significance of the data. For non-normally distributed continuous numerical variables, they were expressed as median and interquartile range (IQR), and the Mann–Whitney U test was used to analyze the significance of the data. For qualitative data, frequencies or percentages (%) were used to represent the data, and significance analysis was conducted using the chi-square test and Fisher’s exact test. We performed a correlation analysis on the positive imaging parameters in patients with active acromegaly. For continuous numerical variables that followed a normal distribution, Pearson test was used, while for non-normally distributed continuous numerical variables or categorical variables, Spearman test was applied. The comparison threshold with significant difference in data was set as *P* < 0.05.

## Results

### Clinical characteristics of enrolled subjects

In the present study, 54 subjects were enrolled, including 27 active acromegaly patients and 27 health subjects with matched age, gender, BMI and BSA (Table 1). The mean age of active acromegaly patients was 40.22 ± 11.14 years, of whom 55.56% were female, with a mean BMI of 25.53 ± 3.80 kg/m^2^. Significantly elevated blood pressure (including SBP and DBP) was observed in active acromegaly patients. 33.3% of active acromegaly patients suffer from hypertension, and all of them received antihypertensive medication. In both groups, there were no significant differences in fasting blood glucose (FBG), uric acid (UA), cholesterol (TC), triglycerides (TGs), high-density lipoprotein cholesterol (HDL-C) and low-density lipoprotein cholesterol (LDL-C) levels. Among patients with active acromegaly, 22.2% had diabetes mellitus, with 18.5% of them taking hypoglycemic medications, and 44.5% had hyperlipidemia, with 37.04% of them taking lipid-lowering medications. In the acromegaly group, the mean GH and IGF-1 are 19.5 ± 33.3 μg/L and 468.5 ± 154.8 ng/mL, respectively. Of these, 44.4% received surgery, 7.4% received radiotherapy, and 33.3% received drug treatment. 51.9% of patients received combined treatments (≥2 treatment modalities, such as surgery + radiotherapy and surgery + medication). Among patients receiving medication-only treatment, 33.3% were treated with dopamine receptor agonists alone, 44.4% with somatostatin analogues alone, and 22.2% with a combination of the two drugs.

**Table 1:** Clinical and demographic characteristics of acromegaly patients and controls.

	Control group	Acromegaly group	*P* value
	*n* = 27	*n* = 27	
Age (years)	33.6 ± 13.6	40.2 ± 11.1	0.054
Sex (female)	48.2% (13)	55.6% (15)	0.297
Height (cm)	167.7 ± 11.4	167.6 ± 11.8	0.983
Weight (kg)	71.5 ± 15.8	72.3 ± 15.4	0.855
BMI (kg/m^2^)	25.4 ± 5.1	25.5 ± 3.8	0.906
BSA (m^2^)	1.8 ± 0.2	1.8 ± 0.3	0.878
SBP (mmHg)	115.6 ± 10.3	127.0 ± 14.8	**0.002**
DBP (mmHg)	74.2 ± 8.0	81.4 ± 11.9	**0.014**
FBG (mmol/L)	4.82 ± 0.54	5.81 ± 1.98	0.085
UA (umol/L)	363.7 ± 150.4	347.5 ± 126.0	0.733
TC (mmol/L)	4.61 ± 0.89	4.67 ± 0.80	0.870
TG (mmol/L)	1.67 ± 1.08	1.24 ± 0.46	0.347
HDL-C (mmol/L)	1.09 ± 0.21	1.22 ± 0.26	0.218
LDL-C (mmol/L)	3.00 ± 0.63	2.85 ± 0.56	0.558
GH (μg/L)	—	19.5 ± 33.3	—
IGF-1 (ng/mL)	—	468.5 ± 154.8	—
Surgery history (%)	—	44.4	—
Radiotherapy history (%)	—	7.4	—
Drug history (%)	—	33.3	—
DAs (%)	—	33.3	—
SSA (%)	—	44.4	—
DAs + SSA (%)	—	22.2	—
Combined treatments (%)*	—	51.9	—
Treatment-naive (%)	—	48.1	—
Relapse after remission (%)	—	29.6	—
Persistent activity (%)	—	22.3	—

Bold indicates statistical significance, *P* < 0.05. Abbreviations: BMI, body mass index; BSA, body surface area; SBP, systolic blood pressure; DBP, diastolic blood pressure; FBG, fasting blood glucose; UA, uric acid; HBA1c, glycated hemoglobin type A1c; TC, total cholesterol; TGs, triglycerides; HDL-C, high-density lipoprotein cholesterol; LDL-C, low-density lipoprotein cholesterol; GH, growth hormone; IGF-1, insulin-like growth factor-1; SSA, somatostatin analogues; DAs, dopamine receptor agonists; Combined treatments*, patients who have undergone two of the three treatment modalities (surgery, medication or radiotherapy).

### Conventional echocardiographic parameters

There was no difference of LVEF between the control group and acromegaly group, both of which were in normal range (59.11 ± 4.80 vs 60.30 ± 3.65, *P* = 0.312). Patients with active acromegaly presented with higher left atrial volume index maximum (22.6 ± 6.3 vs 29.9 ± 10.9, *P* = 0.003), tricuspid regurgitation velocity (1.8 ± 0.5 vs 2.1 ± 0.3, *P* = 0.007), interventricular septal thickness (8.3 ± 1.3 vs 10.4 ± 2.3, *P* < 0.001), posterior wall thickness (7.6 ± 1.0 vs 9.0 ± 1.7, *P* < 0.001), biplane end-diastolic volume (78.3 ± 16.1 vs 92.3 ± 28.6, *P* = 0.032) and biplane end-systolic volume (30.9 ± 6.5 ± 4.80 vs 38.3 ± 14.5, *P* = 0.021). Moreover, the E wave (84.7 ± 18.7 vs 66.9 ± 16.3, *P* = 0.001) and side wall E′ (14.7 ± 3.4 vs 12.5 ± 4.0, *P* = 0.036) were significantly decreased in the acromegaly group (Table 2).

**Table 2:** Conventional echocardiographic parameters in controls and acromegaly patients.

Item	Control group	Acromegaly group	*P* value
	*n* = 27	*n* = 27	
HR (bpm)	70.7 ± 12.1	72.9 ± 14.6	0.549
BiplaneEF (%)	60.3 ± 3.7	59.1 ± 4.8	0.312
S’ (cm/s)	13.1 ± 1.7	12.3 ± 1.5	0.083
Tricuspid regurgitation velocity (cm/s)	1.8 ± 0.5	2.1 ± 0.3	**0.007**
LAVimax (mL)	22.6 ± 6.3	29.9 ± 10.9	**0.003**
IVST (mm)	8.3 ± 1.3	10.4 ± 2.3	**0.000**
PWT (mm)	7.6 ± 1.0	9.0 ± 1.7	**0.000**
LVEDD (mm)	46.0 ± 3.4	48.1 ± 5.6	0.116
LVESD (mm)	29.0 ± 3.4	29.7 ± 4.6	0.524
BiplaneEDV (mL)	78.3 ± 16.1	92.3 ± 28.6	**0.032**
BiplaneESV (mL)	30.9 ± 6.5	38.3 ± 14.5	**0.021**
BiplaneSV (mL)	47.4 ± 10.7	54.0 ± 15.2	0.071
BiplaneCO (L/min)	3.4 ± 0.7	3.8 ± 1.1	0.119
E (m/sec)	84.7 ± 18.7	66.9 ± 16.3	**0.001**
A (m/sec)	64.0 ± 15.7	62.0 ± 16.1	0.668
E/A	1.4 ± 0.5	1.2 ± 0.5	0.097
Interventricular septal E′ (cm/sec)	10.6 ± 2.8	9.4 ± 3.1	0.129
Interventricular septal E/E′	8.3 ± 2.1	7.6 ± 2.4	0.257
Side wall E′ (cm/sec)	14.7 ± 3.4	12.5 ± 4.0	**0.036**
Side wall E/E′	6.0 ± 1.6	5.8 ± 1.6	0.646
Mean E/E′	7.1 ± 1.7	6.7 ± 1.8	0.461

Bold indicates statistical significance, *P* < 0.05. Abbreviations: HR, heart ratio; IVST, interventricular septal thickness; PWT, posterior wall thickness; LVEDD, left ventricular end-diastolic diameter; LVESD, left ventricular end-systolic diameter; EDV, end-diastolic volume; ESV, end-systolic volume; SV, stroke volume; CO, cardiac output; EF, ejection fraction; LAVimax, maximum left atrial volume; E, mitral early diastolic velocity; A, mitral late diastolic velocity; E′, early diastolic myocardial peak velocity lateral annulus; S′, tricuspid annular systolic motion velocity.

### Myocardial strain parameters

**Figure 1:**
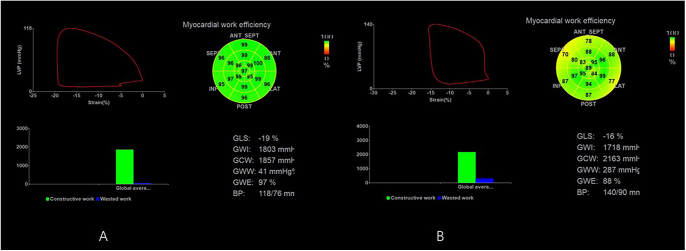
Representative speckle-tracking echocardiography of a matched healthy control (A) and an active acromegaly patient (B). Pressure–strain loops and corresponding bull’s-eye maps illustrate segmental contributions to global MW. The patient exhibits elevated GWW (287 mmHg%) and reduced GWE (88%) versus the control (GWW = 41 mmHg%; GWE = 97%), indicating early systolic dysfunction despite preserved left-ventricular ejection fraction.

**Figure 2:**
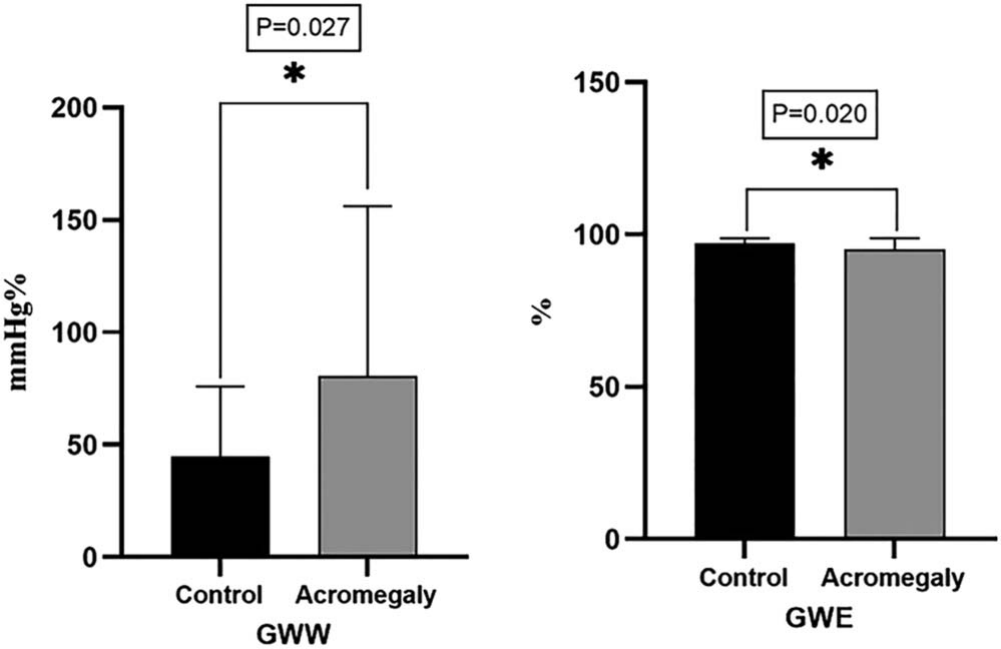
Comparison of GWW and GWE between control group and active acromegaly group. GWW was significantly higher in the acromegaly group than in controls (*P* = 0.027). GWE was significantly lower in the acromegaly group than in controls (*P* = 0.027).

The GWW (44.8 ± 31.1 vs 80.6 ± 75.6 mmHg%, *P* = 0.027) was significantly increased, and the GWE (97.0 ± 1.8 vs 95.0 ± 3.8%, *P* = 0.020) was significantly decreased in patients with active acromegaly (Table 3, Figs 1 and 2). However, the mean GLS was similar between the control group and acromegaly group (Table 3). In addition, pairwise comparisons between subgroups also showed no significant differences in active acromegaly (Supplementary Table S1 (see section on Supplementary materials given at the end of the article)).

**Table 3:** Myocardial parameters and demographic characteristics in acromegaly patients and controls.

	Control group	Acromegaly group	*P* value
	*n* = 27	*n* = 27	
GWI (mmHg%)	1,620.4 ± 270.8	1,534.4 ± 334.2	0.304
GCW (mmHg%)	1,849.3 ± 304.0	1,846.4 ± 375.9	0.975
GWW (mmHg%)	44.8 ± 31.1	80.6 ± 75.6	**0.027**
GWE (%)	97.0 ± 1.8	95.0 ± 3.8	**0.020**
GLS (%)	−18.3 ± 2.4	−17.4 ± 2.9	0.514

Bold indicates statistical significance, *P* < 0.05. Abbreviations: GWI, global work index; GCW, global constructive work; GWW, global wasted work; GWE, global work efficiency; GLS, global longitudinal strain.

### Correlation analysis of GWW and GWE

In acromegaly patients, GWW was positively correlated with age (*r* = 0.49, *P* = 0.009) and 1.5 × ULN IGF-1 (*r* = 0.47, *P* = 0.049) and negatively correlated with hypertension history (*r* = −0.46, *P* = 0.016). GWE was negatively correlated with age (*r* = −0.41, *P* = 0.032) and 1.5 × ULN IGF-1 (*r* = −0.50, *P* = 0.033) and positively correlated with hypertension history (*r* = 0.45, *P* = 0.019). They were both not significantly associated with sex, disease duration, GH and treatment history (Tables 4 and 5).

**Table 4:** Correlation analysis of GWW.

	Correlation analysis	
	*R* value	*P* value
Age	0.49	**0.009**
Sex	0.70	−0.079
Disease duration	0.30	0.126
GH	0.01	0.957
>1.5 × ULN IGF-1	0.47	**0.049**
Hypertension history	−0.46	**0.016**
Treatment history		
Surgery	0.23	0.256
Drug	0.08	0.700
Radiotherapy	−0.07	0.747
Surgery + drug	−0.22	0.268
Surgery + drug + radiotherapy	−0.03	0.872

Bold indicates statistical significance, *P* < 0.05. Abbreviations: GWW, global wasted work; GH, growth hormone; IGF-1, insulin-like growth factor-1; ULN, upper limit of normal.

**Table 5:** Correlation analysis of GWE.

	Correlation analysis	
	*R* value	*P* value
Age	−0.41	**0.032**
Sex	0.17	0.403
Disease duration	−0.25	0.202
GH	0.01	0.957
>1.5 × ULN IGF-1	−0.50	**0.033**
Hypertension history	0.45	**0.019**
Treatment history		
Surgery	0.25	0.217
Drug	−0.26	0.185
Radiotherapy	0.11	0.584
Surgery + drug	0.07	0.723
Surgery + drug + radiotherapy	0.07	0.719

Bold indicates statistical significance, *P* < 0.05. Abbreviations: GWE, global work efficiency; GH, growth hormone; IGF-1, insulin-like growth factor-1; ULN, upper limit of normal.

## Discussion

In the active acromegaly population, more sensitive parameters are required to assess cardiovascular function due to the insidious nature of this disease. The MW index has been confirmed in previous studies for its sensitivity in detecting early myocardial systolic dysfunction (22, 23). In the present study, we found that GWW was significantly increased and GWE was significantly decreased in active acromegaly patients, both of which showed good sensitivity in detecting subclinical myocardial systolic dysfunction. In addition, we found that both GWW and GWE were significantly correlated with 1.5 × ULN IGF-1, suggesting that this threshold may be a key cutoff point for identifying patients at high cardiovascular risk. Our findings facilitate the monitoring of early cardiac structural and functional abnormalities in active acromegaly patients, guide timely clinical interventions and prevent the occurrence of adverse cardiovascular events.

For decades, cardiovascular disease and changes in cardiac structure and function in patients with acromegaly have been major indicator of focus. Echocardiography remains a cornerstone for the cost-effective evaluation and monitoring of these changes. In the present study, acromegaly patients had increased IVST and PWT values, decreased E value ventricular wall diastolic function and higher proportion of myocardial hypertrophy. Moreover, ventricular diastolic function in acromegaly patients is significantly related to whether they have received treatment (24). Clayton *et al.* found that, after the disease was controlled, the myocardial diastolic function could be restored to a certain extent, and the increase in left ventricular mass index (LVMi) in patients with acromegaly decreased GH (25). Compared with diastolic dysfunction, left ventricular systolic dysfunction is closely related to the incidence of cardiovascular diseases (heart failure, myocardial infarction, cardiac interventional surgery, etc.) and cardiovascular mortality, so it is more important in clinical practice.

Previous studies have suggested that left ventricular systolic dysfunction will lead to poor prognosis and increased mortality in patients with cardiomyopathies such as arrhythmogenic cardiomyopathy (26), dilated cardiomyopathy (27), hypertrophic cardiomyopathy (28) and restrictive cardiomyopathy (29). Compared with the traditional echocardiographic index LVEF, GLS measured by cardiac strain and MW indices can reflect early subclinical myocardial systolic dysfunction earlier and more sensitively (30, 31, 32), which were also validated in the previous research (18). We demonstrated that patients with Cushing’s syndrome exhibited an early, asymptomatic reduction in left ventricular GLS despite a preserved ejection fraction (18). However, in the present study, the indicators for evaluating myocardial contractile function, whether LVEF or GLS, did not show significant statistical difference, which were consistent with previous studies (33, 34). MW parameters (GWW and GWE) exhibited significant differences, revealing their good value in early diagnosis.

This was the first study to analyze MW in acromegaly population (35). Previous research has shown that GWW and GWE have higher sensitivity in detecting patients with preserved GLS but impaired myocardial mechanics, such as hemodynamic load abnormalities and myocardial energy abnormalities, which further indicates that MW parameters have higher sensitivity in detecting subclinical myocardial systolic dysfunction in patients with normal GLS (36, 37). Due to the asynchronicity of myocardial hypertrophy in patients with acromegaly, ventricles perform work under different pressures during myocardial contraction, resulting in a global myocardial contraction asynchrony. This kind of non-synchronous stretching will cause part of the work done by some segments of the myocardium during systole to be consumed in the stretching of other segments, resulting in a significant increase in GWW and ultimately a significant decrease in GWE. Meanwhile, GCW (indicating the useful work that promotes myocardial contraction and relaxation) and GWI (indicating the overall work of the myocardium) also decreased in acromegaly but did not show significant statistical difference. Based on the existing literature, it can be inferred that GWW and GWE are potentially more sensitive indicators of left ventricular remodeling compared to GCW and GWI (30). Relevant research has found that in patients with hypertension, the abnormal rate of GWI and GCW was only slightly higher than that of healthy controls, while abnormal GWW and GWE could be observed more frequently, and abnormal GWW and GWE were particularly significant in patients with left ventricular remodeling (30, 38, 39). GCW was also significantly decreased and GWW significantly increased in patients with non-obstructive hypertrophic cardiomyopathy, and they found that GCW was also a sensitive parameter of myocardial fibrosis (40, 41), which was further verified in patients with dilated cardiomyopathy (42). The diagnostic value of GCW in myocardial fibrosis is expected to be further explored in acromegaly. Among the 27 patients with active acromegaly, 13 patients (48.1%) had not received any treatment, 8 patients (29.6%) experienced recurrence after remission, and 6 patients (22.3%) failed to attain biochemical control after treatment. However, we found no significant differences in cardiac structure and function among the three groups. We hypothesize that the small sample size may have affected the statistical power, and it is also possible that the remission period was too short, leading to the sustained hypersecretion of GH and IGF-1, which could further impair cardiac function. In subsequent studies, we plan to include a larger sample size to further enhance statistical power.

Furthermore, we investigated the risk factors associated with GWW and GWE. Our findings showed that GWW had a positive correlation with age and 1.5 × ULN IGF-1 and a negative correlation with a history of hypertension. In contrast, GWE exhibited a negative correlation with both age and 1.5 × ULN IGF-1, while it showed a positive correlation with a history of hypertension. We found that the correlations between age and MW parameters (GWE and GWW) were consistent with the progression of acromegaly. The inverse correlations observed between MW parameters (GWW and GWE) and a history of hypertension could be explained by the inclusion of patients from different age groups and stages of the disease, which may result in varying correlation patterns. The health status of hypertensive patients could differ across these subgroups, potentially influencing the outcomes. In addition, our study did not consider the duration of hypertension in the patients, which may be another confounding factor. Future research with a larger sample size and subgroup analysis will be necessary to further explore these relationships and control for potential confounding variables. It was worth noting that both GWW and GWE were significantly correlated with 1.5 × ULN IGF-1, which may be due to the variability of the assay and the fact that some acromegaly patients included in this experiment were treated after comprehensive treatment. Previous studies have shown that compared with acromegaly patients whose IGF-1 level is 1 × ULN, those with active acromegaly and IGF-1 level of 2.2 ± 1.1 × ULN exhibit more significant cardiovascular structural abnormalities (e.g., vascular endothelial dysfunction) (43). Similarly, in the present study, we found that 1.5 × ULN IGF-1 was significantly correlated with MW parameters, which may suggest that 1.5 × ULN can serve as a ‘warning’ cut-point for impaired cardiovascular function. Since MW parameters reflect myocardial contractile function, the correlation between 1.5 × IGF-1 ULN and these MW parameters may indicate that 1.5 × IGF-1 ULN can serve as a key threshold for identifying patients at higher cardiovascular risk. Clinicians should consider performing comprehensive cardiovascular assessments for acromegaly patients with IGF-1 levels above this threshold to mitigate the risk of adverse cardiovascular outcomes, and close monitoring of these patients is recommended to manage potential complications effectively. Due to the relatively small sample size in the present study, a more accurate cutoff value may require a larger sample size to verify in the future.

## Limitations

Our study has several shortcomings. First of all, this was a retrospective, cross-sectional study without follow-up on the patients. Moreover, because acromegaly itself is an extremely hidden and rare disease, there is a lag from the emergence of relevant clinical manifestations to the initial diagnosis, which leads to a deviation in the judgment of the course of acromegaly patients. In addition, the sample size of this study was small, which prevented us from accurately assessing the differences in myocardial function parameters between acromegaly patients and the control group. A larger sample size is needed for further verification. Finally, in the assessment of MW, since systemic arterial pressure measured directly with a cuff represents left ventricular systolic pressure, when there is a difference between systemic arterial pressure and left ventricular pressure (such as aortic stenosis and left ventricular outflow tract patients with obstruction), it will lead to errors. Because this study is a single-center study, the same detection method was used for clinical judgment, the evaluation method and evaluation cutoff point were the same, and the whole study was conducted by the same experimenter. The errors caused by detection methods and heterogeneity among researchers were avoided.

## Conclusion

GWE and GWW are sensitive markers in monitoring left ventricular remodeling and early systolic dysfunction in active acromegaly patients, which may help monitor subclinical myocardial systolic dysfunction and prevent the occurrence of cardiovascular adverse events. Prospective studies with larger sample sizes are warranted to validate the diagnostic value of MW indices for cardiovascular prognosis.

## Supplementary materials



## Declaration of interest

The authors declare that there is no conflict of interest that could be perceived as prejudicing the impartiality of the work reported.

## Funding

This study was supported by Science and Technology Projects in Guangzhou (2023A04J2190), National Natural Science Foundation of China Youth Science Foundation (82201551), and Guangzhou 2023 Basic and Applied Basic Research Project (2023A04J2196). The funders were not involved in study design, the collection and analysis of data, writing of the manuscript or the decision to submit the article for publication.

## Author contribution statement

RH, PYZ and WH were responsible for designing the work, interpreting the results and writing the manuscript; JWJ and YP helped acquire data, perform analysis with constructive discussions and make tables; YBL and HPX revised the manuscript; and FJY and HL designed the work, revised the manuscript and approved the final version. All authors approved this version, have participated sufficiently in the work, and agree to be accountable for all aspects of the work including integrity and accuracy.

## Ethical statement

All research methods were approved by the Medical Ethics Committee of the First Affiliated Hospital of Sun Yat-Sen University, and were in accordance with the 1964 Declaration of Helsinki and its later amendments or comparable ethical standards. The purpose, method and potential risks of the study were explained to all subjects, and all subjects signed an informed consent form.
