# Supplementary material for: Diagnostic and prognostic value of myocardial work indices in evaluating cardiac function damage in active acromegaly patients
Source: Endocr Connect. 2026 Jan 12;15(1):e250478. doi: 10.1530/EC-25-0478 (PMC12809010; doi:10.1530/EC-25-0478)
Supplement: Supplementary file 1 [file supplementary_materials.pdf]

Table S1 Subgroup Comparison of Key Parameters in Active Acromegaly

	Treatment-naïve N=13	Relapse after remission N=8	Persistent activity N=6	$P^{\#}$value	P^{*} value	$P^{\&}$ value
GH(ug/L)	27.8±2.8	41.0±9.6	7.0±5.2	0.623	0.478	0294
IGH-1(ng/mL)	490.3±167.6	386.5±146.0	432.5±98.0	0.135	0.440	0.573
GLS(%)	-17.5±2.8	-18.6±2.6	-15.7±2.8	0.373	0.201	0.062
GWI(mm Hg%)	1606.0±332.5	1532.1±370.9	1382.3±285.9	0.628	0.189	0.416
GCW(mm Hg%)	1945.0±321.2	1532.1±370.9	1382.3±285.9	0.645	0.070	0.191
GWW(mm Hg%)	107.8±98.4	64.8±32.5	42.5±30.4	0.202	0.083	0.578
GWE(%)	94.2±4.8	95.4±2.8	95.0±3.8	0.488	0.230	0.594
IVST(mm)	10.1±1.2	9.6±1.3	11.8±4.1	0.634	0.131	0.082
PWT(mm)	8.8±1.0	8.6±1.5	9.8±2.6	0.820	0.199	0.179

Abbreviations: GH, growth hormone; IGF-1, insulin like growth factor-1; GWI, global work index; GCW, global constructive work; GWW, global wasted work; GWE, global work efficiency; GLS, global longitudinal strain; IVST, interventricular septal thickness; PWT, posterior wall thickness; $P^{\#}$, “Without treatment” vs “Relapse after remission”; P^{*}, “Without treatment” vs “Persistent activity”; $P^{\&}$, “Relapse after remission” vs “Persistent activity”.
